# ML Approach to Improve the Costs and Reliability of a Wireless Sensor Network

**DOI:** 10.3390/s23094303

**Published:** 2023-04-26

**Authors:** Mehmet Bugrahan Ayanoglu, Ismail Uysal

**Affiliations:** Department of Electrical Engineering, University of South Florida, Tampa, FL 33620, USA

**Keywords:** machine learning, wireless sensor networks, time series, cold chain, transportation, convolutional neural networks

## Abstract

Temperature-controlled closed-loop systems are vital to the transportation of produce. By maintaining specific transportation temperatures and adjusting to environmental factors, these systems delay decomposition. Wireless sensor networks (WSN) can be used to monitor the temperature levels at different locations within these transportation containers and provide feedback to these systems. However, there are a range of unique challenges in WSN implementations, such as the cost of the hardware, implementation difficulties, and the general ruggedness of the environment. This paper presents the novel results of a real-life application, where a sensor network was implemented to monitor the environmental temperatures at different locations inside commercial temperature-controlled shipping containers. The possibility of predicting one or more locations inside the container in the absence or breakdown of a logger placed in that location is explored using combinatorial input–output settings. A total of 1016 machine learning (ML) models are exhaustively trained, tested, and validated in search of the best model and the best combinations to produce a higher prediction result. The statistical correlations between different loggers and logger combinations are studied to identify a systematic approach to finding the optimal setting and placement of loggers under a cost constraint. Our findings suggest that even under different and incrementally higher cost constraints, one can use empirical approaches such as neural networks to predict temperature variations in a location with an absent or failed logger, within a margin of error comparable to the manufacturer-specified sensor accuracy. In fact, the median test accuracy is 1.02 degrees Fahrenheit when using only a single sensor to predict the remaining locations under the assumptions of critical system failure, and drops to as little as 0.8 and 0.65 degrees Fahrenheit when using one or three more sensors in the prediction algorithm. We also demonstrate that, by using correlation coefficients and time series similarity measurements, one can identify the optimal input–output pairs for the prediction algorithm reliably under most instances. For example, discrete time warping can be used to select the best location to place the sensors with a 92% match between the lowest prediction error and the highest similarity sensor with the rest of the group. The findings of this research can be used for power management in sensor batteries, especially for long transportation routes, by alternating standby modes where the temperature data for the OFF sensors are predicted by the ON sensors.

## 1. Introduction

The wireless environmental monitoring applications in today’s cold chain present a range of challenges, including the cost of the hardware implementation, limitations due to the high power consumption of sensors, and the general ruggedness requirements of the environment [1,2]. There is a common thread when it comes to tackling both challenges using data-driven approaches, including statistical analysis and machine learning. The underlying assumption of this study is that the physical dynamics and environmental factors of the transport container remain relatively unchanged during the training and testing process. However, in a real-life application, the wireless network first must be trained on the specifics of the transportation container without making too many modifications. The volatility of the estimation models in wireless networks itself is a different kind of research question which is not in the scope of this paper. This paper presents a detailed study of a novel real-life dataset where a sensor network is implemented to monitor environmental temperatures at different locations inside a temperature-controlled shipping container across different scenarios.

Specifically, the possibility of predicting one or more locations inside the container in the absence or breakdown of a logger placed in that location is explored using combinatorial input–output settings where 1016 algorithms are exhaustively trained, tested, and validated. The statistical correlations between different loggers and logger combinations are studied to identify a systematic approach to finding the optimal setting and placement of loggers under a cost constraint.

At its core, wireless sensor analytics represent a form of multivariate time series analysis, as the data being collected by the sensor are represented temporally across one or more dimensions. Yang et al. [3] suggest that the time series analysis presents unique challenges compared to more static data analytics problems such as image and pattern recognition. These challenges are represented both at the algorithm level as well as the hardware level, where malfunctioning sensors can generate incorrect or missing data.

In this paper, we concentrate on a specific dataset that represents temperature distributions inside shipping containers for perishable food products (in this case strawberries). It has been demonstrated that 50% of harvested strawberries are wasted due to inadequate temperature monitoring and control [4]. An intelligent setup of a wireless temperature sensor network can alleviate some of this waste with smart distribution practices such as first-expired-first-out instead of first-in-first-out. Nevertheless, the cost of installation is prohibitive to most industries, including fresh produce, which limits the number of sensors that can be utilized in a shipping container [4,5].

There are benefits to replacing node data in unexpected situations of such a sensor network through the use of machine learning and artificial intelligence methods. These benefits include improving monitoring accuracy and resolution, preemptively recovering from sensor malfunctions, and intelligent power management to reduce regular battery maintenance. Time series prediction and analysis have been discussed and articulated in the literature for many decades across different field applications [6]. Methods including Auto-Regressive Moving Average (ARMA) [7], Auto-Regressive Integrated Moving Average (ARIMA) [8], Seasonal ARIMA [9], exponential smoothing [10], and Vector Autoregression (VAR) [11] are parametric models which require prior statistical knowledge on the time series data, which may not be feasible for practical applications. Non-parametric methods for time series modeling and prediction including neural networks [12,13] have been successfully applied to different time series modeling and forecasting applications [14,15,16,17].

A popular modification of neural networks for time series analysis is the recurrent neural network (RNN) topology [18,19]. Specifically, long short-term memory (LSTM) [20] and gated recurrent unit (GRU) [21] networks build upon the conventional RNN to address the drifting gradient problem [22,23] and have been applied to both univariate [24] and multivariate time series applications [25,26].

The main architecture used in our study utilizes convolutional neural networks (CNNs), more commonly used in the image processing field. LeCun et al. used images of hand-written numbers to learn the convolution kernel coefficients [27]. This approach became the foundation of modern computer vision. However, CNNs are not limited to handling images. Although CNNs for deep learning applications were developed for two-dimensional image data, they can be used to model univariate time series forecasting problems. CNNs [28] have successfully been implemented across many domains, including time series forecasting [29,30] and classification [31,32]. In this research, we use a single-dimensional univariate system for the given time series prediction problem.

To capture and understand the multiple related time series specifications of this novel data, before training in our neural network model, we analyze the dataset using algorithms, such as Pearson’s correlation coefficient and dynamic time warping, to determine indications of the time series to be used in subsequent time series applications.

Our findings show that even under different and incrementally higher cost constraints, one can use empirical approaches such as neural networks to predict temperature variations in a location with an absent or failed logger within a margin of error comparable to the manufacturer’s specified sensor accuracy. Our findings also show that using correlation coefficients and time series similarity measurements, one can identify the optimal input–output pairs for the prediction algorithm reliably under most instances.

This study has four different contributions to the science of using wireless sensor networks in practical settings which are as follows:

1. This study presents a real-life application of a wireless sensor network used to monitor different locations inside temperature-controlled shipping containers during both short- and long-distance travels.
2. This study explores the possibility of sensor replacement for either power-management or cost-reduction purposes using the complete combinatorial analysis across the sensor network by exhaustively training, testing, and validating 1016 different machine learning models to search for the best model.
3. This study finds that even under incrementally higher cost and power constraints, prediction within the margin of error for sensor accuracy is not only possible but readily achievable.
4. Finally, this study introduces a systematic approach to find the optimal setting and placement of sensor nodes using statistical and temporal similarity measures such as correlation coefficients and discrete time warping.

The paper is organized as follows: Section 2 describes the procedure of the collection of the data and the novel univariate time series. A brief explanation of univariate time series and the novel data is also presented in this section. Section 3 introduces the approach of the research in detail. Statistical methods to capture meaning out of the data are described in Section 3.1, followed by the processes to prepare the data for the neural network model in Section 3.2. Section 3.3 and Section 3.4 detail the generation and application of the model. The results are presented in Section 4, followed by the conclusions in Section 5.

## 2. Datasets: Cold Chain Univariate Temperature Time Series

**Figure 1:**
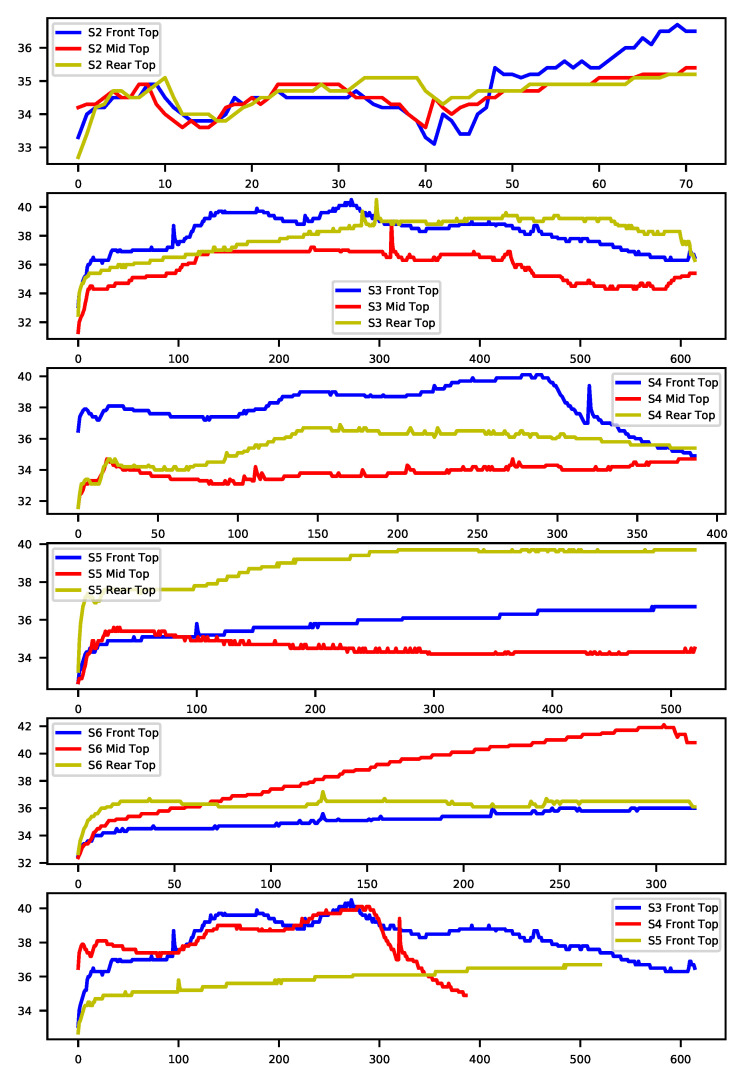
Temperature profiles of multivariate time series data from pre-cooling to the end of transportation, S2–S6. Comparison of front top sensors of S3–S5 transportations to represent the different characteristics of transportation route data.

Formation of the dataset: the temperature and humidity levels of the goods in cold chain transportation are monitored via cold chain data loggers for quality insurance purposes. In our project, DeltaTrak’s Reusable Real-Time-Logger (RTL) was used to monitor and log the temperature, timestamp, and corresponding location data. The data were collected from five different shipments that have nine sensors placed in different locations within the transportation medium, the container. Three pallets are placed in the container, one up front, one in the middle, and one towards the rear side. Each of these pallets has three sensors placed vertically, one at the bottom of the pallet, one toward the middle, and one on the top of the pallets, which is expected to have a higher airflow. The data loggers record the data at 15-min intervals for the duration of the transportation route. Although the ideal transportation and storage temperatures are in the range of (32 °F), due to issues such as insulation problems, doors opening and closing during the loading and unloading stages, and irregular air circulation, sudden, unexpected variations in the temperature profiles occur. These variations can be seen in Figure 1, the representation of the univariate dataset used in this research, which is formed by combining data from the five different transportation routes.

A time series $X_{t}$ of size *n* is defined as a collection of data points measured sequentially over equally spaced time intervals. i.e., $X_{t}$ = $\left(x_{1},x_{2},\ldots ,x_{n}\right)$, where $x_{t}$ ∈R is an observation at time t. The novel data presented are a univariate time series since observations are recorded over a single variable. In total, our novel dataset consists of 40 time series (from eight sensor locations within the containers of each of the five shipments). The lengths of the time series vary due to the number of observations recorded for different lengths of the transportation routes and the sensor start/stop times. Table 1 displays the transportation routes and collected data lengths accordingly.

## 3. Materials and Methods

**Figure 2:**
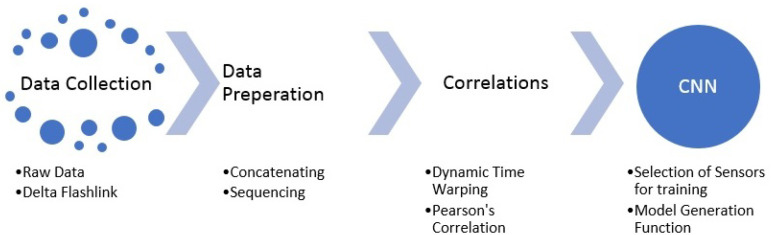
Flowchart of the research approach.

An overview, block diagram of the project is shown in Figure 2. First, the collected data need to be processed to create sequences to generate the necessary input structure to be fed into our model architecture. After generating the sequences for datasets collected from each of the transportation routes, the sequences were concatenated. At this point, we had generated 9 input matrices—created by concatenated sequences—and 9 output arrays that correspond to the sensor locations. The goal was to train and test the ML algorithms, depending on the number of sensors, with combinations of different sensor locations used to predict the values of other sensor locations in case of a faulty sensor or missing data.

We tested and compared the performance of different neural network models to their loss functions. Once the model was selected, we used the sensor data with different amounts of sensors used as inputs, and used different sensor locations to create a variety of combinations to test and predict the temperature levels of the rest of the sensors which were not used in training.

### 3.1. Sensor Data Correlations

The generations of the individual models, model training, and testing phases can be time- and memory-expensive. Before we applied deep learning methods, to give us a better understanding of the univariate data, their distribution, and correlations within the sensor data depending on locations, we decided to apply statistical techniques. We generated Pearson’s correlations matrix and also looked at the dynamic time warping—the statistical distances between data from each of the sensors.

**Figure 3:**
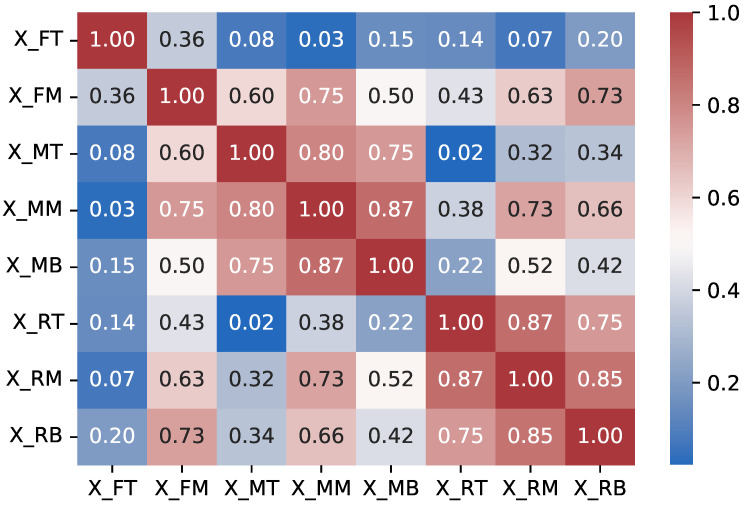
Pearson’s correlation heat map.

Pearson’s correlation coefficient provides a measure of linear similarity between two variables by simply attempting to plot a function to best fit the data of these two variables. The coefficient of correlation dictates the relativity of the data points to this function plot. Our correlation matrix is given in Figure 3, which displays Pearson’s correlations between the sensor location temperature values data. The correlations can take a range of values between −1 and 1. Negative numbers indicate a negative correlation, while positive numbers indicate a positive. A value of 0 indicates there is no correlation between the variables, and a value of 1 indicates that the variables are simply the same. Using Pearson’s correlation coefficients, we looked at the similarities and differences between different sensor locations’ temperature series inside different transportation containers. For instance, X_MB and X_MM have a correlation value of 0.87. This could be explained by the locations of these sensors being close to each other. For these two locations, since they are highly correlated in data, we can simply say that X_MB and X_MM can be used to represent one another. On the other hand, the correlation between X_FT and X_RB is 0.2, which is also expected since the two locations are further apart from each other. Table 2 tells us that if one was to use Pearson’s correlation matrix method to understand, expect, or try to predict the temperature levels of the other sensor locations, only depending on data from a single sensor, X_MM, and secondly X_FM, provide the best results, since these are the most correlated to the rest of the sensors in summation. It should be pointed out that, according to Pearson’s correlation results, the data from the middle sensors (X_MM, X_FM, and X_RM) seem to have more correlation, providing better information on the rest of the data.

The dynamic time warping (DTW) algorithm considers two sequences that are usually temporal, and provides us with a similarity measure between the sequences. DTW mainly warps the time axis of the profiles to be compared, to achieve a better alignment by creating a matrix where the matrix elements are the Euclidean distances between the temperature data points. DTW has been introduced for speech applications by Vintsyuk et al. [33]. The DTW table given in Table 3 represents the average DTW distance of each sensor location’s data to the data from the rest of the sensor locations. As we can see, the sensor locations that have the smallest DTW distance from the rest of the sensor data values are the sensors placed in the middle of the pallets. Sensors X_MM and X_FM have the smallest DTW distance and are the best sensors to give us information on the rest of the sensors. Again, similar to the results we have observed using Pearson’s correlation method, it should be pointed out that according to DTW results, as seen in Table 3, the data from the middle sensors (X_MM, X_FM, and X_RM) seem to have less distance to the rest of the data collected from other sensor locations, hence providing better information on the rest of the data.

### 3.2. Dataset Preprocessing

The training of the univariate CNN model requires sequences of temperature readings as input. The model then maps these input sequences to corresponding output observations. For this purpose, we needed to create the input sequences of temperature observations and transform these into multiple samples or instances from which the model could learn.

**Figure 4:**
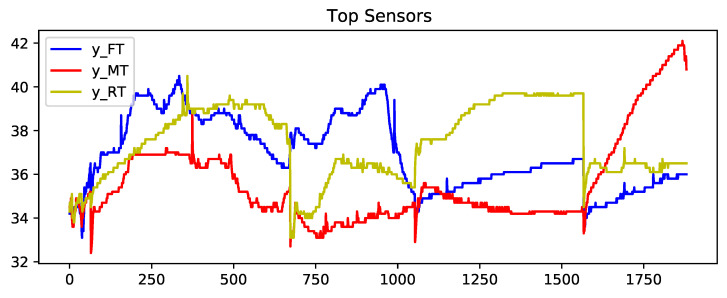
Output arrays: sequenced temperature profiles of top sensors data from all transportations concatenated.

**Figure 5:**
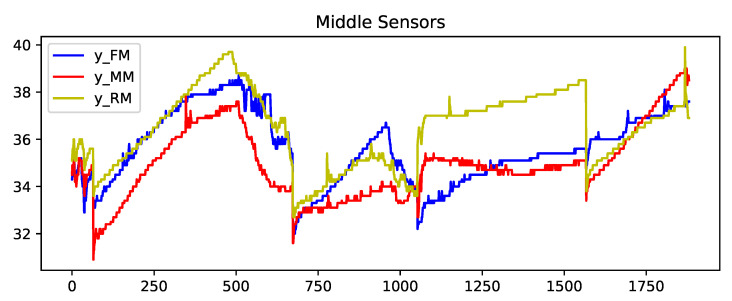
Output arrays: sequenced temperature profiles of middle sensors data from all transportations concatenated.

**Figure 6:**
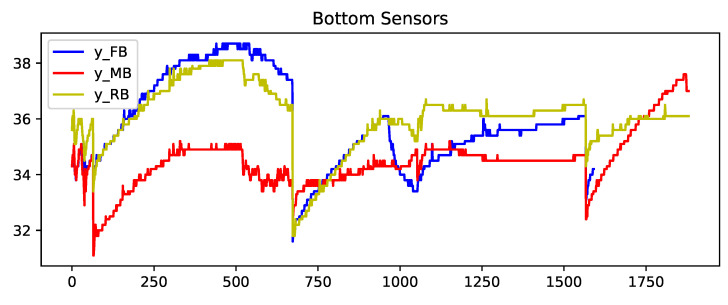
Output arrays: sequenced temperature profiles of bottom sensors data from all transportations concatenated.

Figure 4, Figure 5 and Figure 6 show the concatenated temperature readings from all of the transportation routes. This is presented to display the relationship between the closely located sensors. The temperature values in Fahrenheit are represented on the *Y*-axis and the *X*-axis has the observations (a total of 1920 observations) recorded in 15-min intervals.

**Figure 7:**
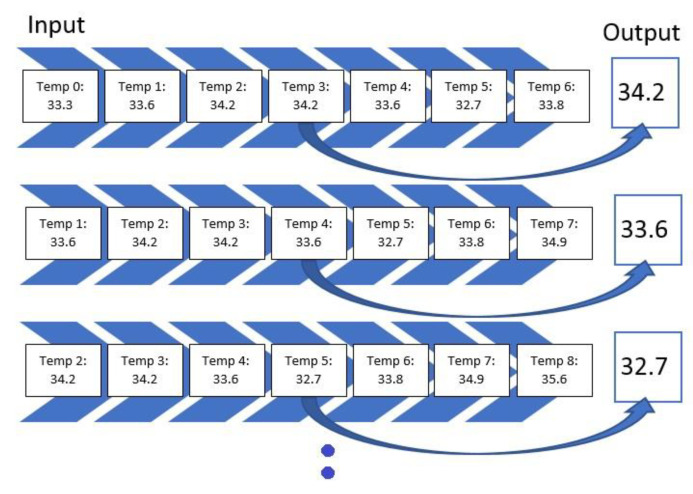
The formation of X_FT and y_FT from the front-top location sensor data time series.

Sequencing is performed by creating an input array of seven consequent temperature readings as instances. Figure 7 displays the creation of the first three sequences. The first sequence starts from the observation at t = 0, and the next sequence starts from the observation at t = 1, and so on. By adding the consecutive sequences in row form, we start forming our input matrix.

The third data point of each of the sequences created is isolated and then appended to another array to form the output vector. Similarly, for the very first output, the label of the first sequence is the third temperature value, and the next one is the fourth. As the input sequence set iterates, the first data and the output label of the sequence shift.

Algorithm 1, Sequence Creation, generates the matrix of X (input) sequences and Y (output) arrays for each of the sensor locations.

To create one input matrix for each of the sensor locations across the 5 different transportation batches, we needed to concatenate these time series. Concatenating the univariate time series data of each sensor location from different transportation batches before forming sequences would have created problems. Incorrect sequences would have been created at the time instances where we switched from one time series to another. Simply, the last observations of one time series and the first observations of the next would combine and create an artificial, incorrect sequence. Therefore, we created 7 input sequences for 5 individual transportation dataset batches for the particular sensor location, and then combined the sequences to form overall data matrices.
**Algorithm 1** Sequence Creation1: **for** Univariate Temp Data, i = 0,1,2,… Sequence Length **do**2:    **if** i + Sequence Length = Temp Data length (End of Data?) **then**3:          Break4:    **end if**5:    X = Array; i, i + 1,… i + Sequence Length6:    Y = Value; i + 37:    X data; append to matrix formation8:    Y data; append to column formation9: **end for**

### 3.3. Model Generation

The CNN used in the research is a sequential model that consists of a Conv1D layer, MaxPooling, flattening functions, and multiple dense layers. The Keras libraries API (Application Programming Interface) was used in the generation of the neural network model, and Scikit-learn API was used for statistical executions, predictions, and evaluation metric functions. Multiple models with different architectures have been tested and compared in the search for the lowest mean absolute error. For instance, a network model with 64 filters at the first convolutional layer and 50 nodes at the dense layer provided a test MAE result of 1.54. Another model, with 64 filters at the first convolutional layer and 25 nodes at the dense layer, provided an MAE of 2.31. The nodel with 64 filters at the first convolutional layer and 100 nodes at the dense layer provided a 2.08 MAE, the model with 32 filters at the first convolutional layer and 50 nodes at the dense layer provided a 2.24 MAE, and another model with 32 filters at the first convolutional layer and 25 nodes at the dense layer provided an MAE of 3.46. Many models have been trained and tested for this intuitive research to optimize the performance of our dataset and its application. Each of the models were run ten times and MAE performances have been averaged.

To create models with different input shapes, we defined a function called ModelG. The ModelG function uses the parameter “inp_shp” to define the size of the input shape of the first convolutional layer, Conv1D. The purpose of this is to use the defined function to call, create, and fit the model, even if the number of sensors used to train the model varies. In our model, we used the Adam optimizer of the Keras optimizers, with a learning rate of 0.01 [34]. Adam is a gradient-based stochastic optimization method that is readily available in the Keras library. As our cost function, we used the loss function formulated by the mean absolute error.

(1) $$MAE=\frac{\sum_{i=1}^{n}\left|y_{i}-x_{i}\right|}{n}$$

Once the model was created, we saved the initial weights in order to have an average of the cost function value acquired for each training and testing pair run of the model, given the fact that we start from the same weights as a base. Algorithm 2 details the generation of the individual models, initialization of the weights, fitting, and evaluation of the architectures.

### 3.4. Model Fitting and Evaluation Metrics

The goal of the project is to predict missing sensor data or to be able to come up with a temperature monitoring system that requires the use of fewer sensors. Therefore, we need to define an optimal cost efficiency ratio to determine the number of sensors and the corresponding locations to be used for a robust temperature monitoring system. For this purpose, we generated our input matrix—using the sequences created—depending on the number of sensors that will be used in the training of the network.

To assist the reader in understanding how exactly the input is formed and the model is evaluated, the steps of the process for a given number of sensors used (*n* = 2) can be described as follows.

- Number of Sensors = 2. When using two sensors as an input we have 28 combinations of sensors out of eight different sensor locations, C(8,2), (Comb1,Comb2,…Comb28), to make predictions on each of the six remaining sensor location data values.
- Input Dataset = A sample Comb1 input would be the sequences of two sensors concatenated on columns, forming an *n* × 14 matrix, Comb1 = [ X_FT, X_RB ], where each sensor data matrix is *n* × 7.
- Output Vectors = To introduce the output to the network so that the model can be trained for a prediction algorithm, we iterated through each of the remainder sensor locations that were not used in the input combination and evaluated the results for each prediction value. As discussed before, the output vectors are formed by appending the third value of the sequences created, with the size *n* × 1. For a Comb1 input provided above that uses X_FT, X_RB as inputs, the output vectors would be [y_FM, y_FB, y_MT, y_MM, y_MB, y_RM]. These output vectors are individually predicted, and evaluation MAE values are obtained.

Each of the models has an epoch number of 10. An averaging of prediction results is necessary for a better comparison of different ML models. After running each of the models multiple times and seeing an insignificant fluctuation in the prediction results, we decided to keep the number of runs minimal for averaging purposes, 10 runs. Data were split in the ratios of 60%, 20%, and 20% for training, validation, and testing purposes, respectively. The models were separately trained for one, two, three, four, five, six, and seven sensors, combinatorially. Meaning, for instance, that the combinations of two sensors are used to train the model, and prediction performances on the values of the rest of the six sensor locations are compared. For a number of two sensors, each of the two combinations of eight sensor locations C(8,2) were matched to six output labels, resulting in a total of 168 models trained and tested.
**Algorithm 2** Model Generation and Evaluation  1: Generate the CNN Model  2: **Input:** $\Omega_{in\;put}$ = { $X_{i}^{n^{\left[0\right]}}$, $X_{i}^{n^{\left[1\right]}}$, …, $X_{i}^{n^{\left[k\right]}}$ }  3: **Output:** Time series signal that represents sensor data to be predicted.  4: **Save** CNN weights  5: **for** Each Combination in C(X of each sensor, # of sensors) **do**  6:    **for** Y of each sensor **do**  7:       **for** i = 0, 1, 2 …# of runs to average **do**  8:             **Compute** Model Fit  9:             **Compute** Train, optimize (ADAM)10:           **Evaluate** $MAE=\frac{\sum_{i=1}^{n}\left|y_{i}-x_{i}\right|}{n}$11:       **end for**12:       **Compute** avg(MAE, train), avg(MAE, test)13:       **Load** CNN Weights14:    **end for**15: **end for**

Similarly, combinations of three sensors were evaluated based on prediction results of the rest of the five sensor location temperature values, 56 (C(8,3)) input combinations, five output labels for each, a total of 280 models trained and tested.

**Figure 8:**
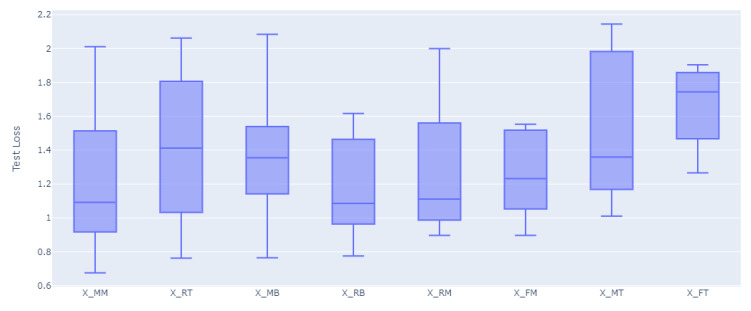
Prediction Loss Distributions—1 to 1 Sensor.

**Figure 9:**
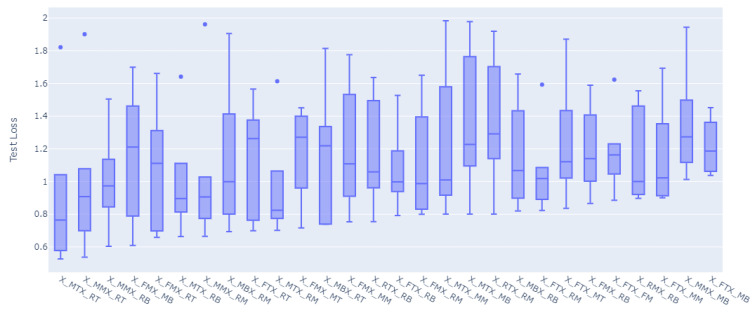
Prediction Loss Distributions—2 to 1 Sensor.

**Figure 10:**
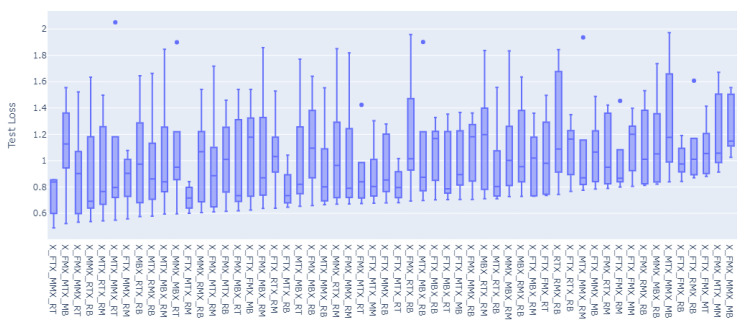
Prediction Loss Distributions—3 to 1 Sensor.

**Figure 11:**
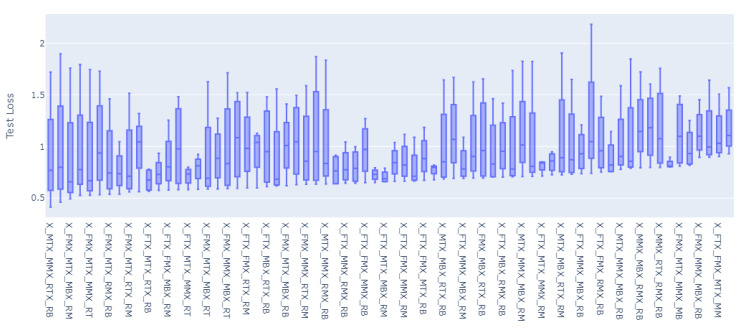
Prediction Loss Distributions—4 to 1 Sensor.

**Figure 12:**
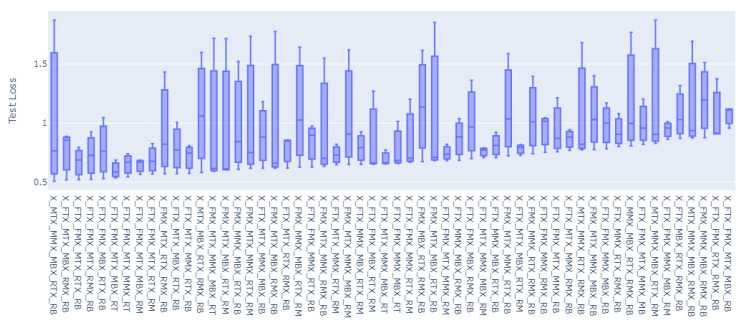
Prediction Loss Distributions—5 to 1 Sensor.

**Figure 13:**
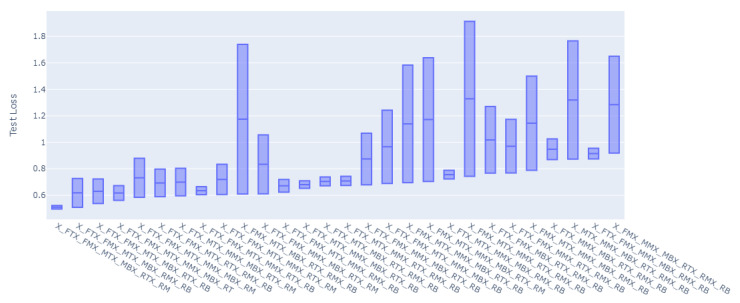
Prediction Loss Distributions—6 to 1 Sensor.

For all of the combinations of the seven sensor locations used for training and prediction for the remainder of the sensor locations, a total of 1016 models were trained and evaluated. The overall loss results, MAE, have been grouped to the inputs sensors used for the model, and the distributions of the prediction results on the rest of the sensors’ values have been graphed accordingly as shown in Figure 8, Figure 9, Figure 10, Figure 11, Figure 12 and Figure 13.

## 4. Results

**Figure 14:**
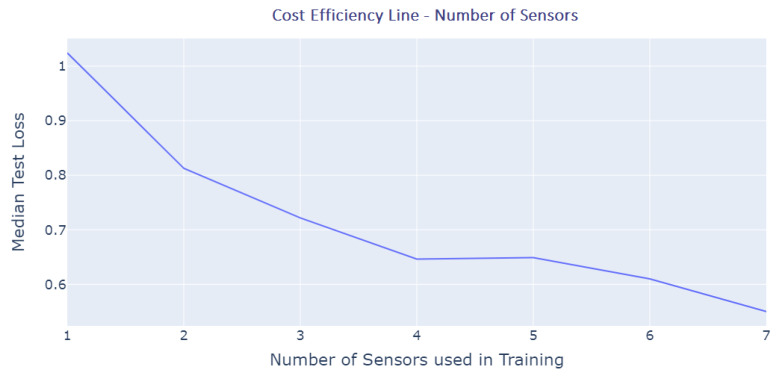
Cost Efficiency Graph.

From the cost efficiency graph, Figure 14, we interpret that as the number of sensors used to predict the values of missing data increases, the prediction loss decreases. On the *X*-axis, one can see the sensor count which indicates the number of sensors used in monitoring the temperature distribution. For instance, 1 indicates using a single sensor to monitor all locations whereas 7 indicates having a sensor in seven locations (and thus effectively having a minimal error). If we look at the derivation of the loss function with the increment in the number of sensors, the largest drop in the loss function values occurred in the first three increments of the sensor count. This means that the return on investment has its biggest margins up until the usage of four sensors. The loss function continues to improve as the number of sensors increases, which is expected, but the improvement in the loss function is much less significant.

**Figure 15:**
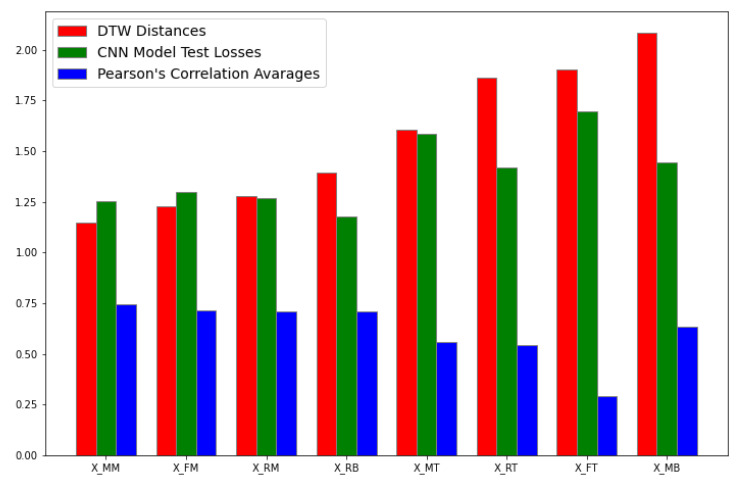
Pearson’s Correlation, DTW, and Test Loss Comparison.

Figure 15 shows the comparison of the results of the statistical techniques used to describe the data and the CNN architecture test loss results. First, comparing the values we gathered from the statistical approaches, Pearson’s correlation and dynamic time warping, we observe a negative correlation, meaning as the DTW values increase, the Pearson’s correlation values decrease. This can be intuitively explained: if two variables have less DTW distance in between, they are more correlated. The loss function results of the CNN model represent a similar trend. As the data sequence becomes less correlated and more distanced from the sensor data of the other sensors, we obtain a larger MAE.

Figure 8, Figure 9, Figure 10, Figure 11, Figure 12 and Figure 13 display the order of the most efficient sensors that provide more accurate prediction performances for the number of sensors used, one, two, three, four, five, and six, respectively. The *X*-axis has the combinations of the sensors used as training to predict the values of the sensors that are not used in training. The box plots display the mean, min, and max values of the MAE results gathered from the testing phases. The vertical lines outside the boxes in each column of the figure represent the outliers for that particular distribution. The order of the plots is from the smallest minimum MAE values obtained to the largest minimum MAE values obtained for each combinatorial training input.

Table 4 gives a summary of the results of the training and testing combinations. For instance, if we were to use one sensor per transportation, the rear-bottom or the middle-middle sensor locations would provide the best results, 1.17 and 1.25, respectively. If we were to use two sensors, the combinations middle-top and rear-bottom or middle-top and rear-middle would give the best prediction results, 0.933 and 0.939 MAE, respectively.

## 5. Discussion and Conclusions

This paper presents a holistic approach to sensor node replacement in wireless sensor networks using statistical time series analysis with Pearson’s correlation or dynamic time warping coupled with a data-driven algorithm in convolutional neural networks. More than a thousand input–output combinations were analyzed and trained methodically to create a cost efficiency curve in a sensor network used to monitor commercial temperature-controlled shipping containers. We demonstrated how computationally inexpensive statistical methods can be used to identify the best sensors for prediction prior to the computationally intensive training of complex machine learning models. With prediction errors as low as 0.98 when using a single sensor to predict the other seven locations, which is less than the manufacturer-reported temperature accuracy, one can utilize the proposed approach for improving the monitoring accuracy and resolution, preemptively recovering from sensor malfunctions and intelligent power management to reduce regular battery maintenance.

Future work will focus on two different trajectories: The first is the automated selection of the best algorithm based on the failure or low-power detection on the wireless sensor networks, defining the ideal power consumption vs. accuracy, and choosing the best number of sensors and the best combination to be used to provide a prediction result to the temperature-control closed-loop system. By this approach, the algorithm can also be incorporated to detect a faulty sensor by comparing predictions, depending on the rest of the sensors, with the actual data the suspected sensor provides. Secondly, it will also investigate other kinds of time series prediction models such as RNN and LSTM, which are more time-intensive to train, but with the help of statistical pre-calculations, this may be accelerated and could potentially yield better results. Furthermore, the algorithm can also be incorporated to detect a faulty sensor by comparing prediction—depending on the rest of the sensors—and the actual data of the suspected sensor.

## Figures and Tables

**Table 1:** Length of temperature time series profiles for all sensors and across all the shipments.

Data Source Abbr.	Transportation Route	Data Length
S2	Plant City, FL—Florida	72
S3	Salinas, CA—Virginia	616
S4	Salinas, CA—South Carolina	388
S5	Salinas, CA—Maryland	522
S6	Plant City, FL—Georgia	322

**Table 2:** The sensor location correlations to the rest of the sensors summed.

Sensor Location Data	Summation of Correlations
X_MM	5.2236
X_FM	5.0009
X_RM	4.9718
X_RB	4.9524
X_MB	4.4288
X_MT	3.9051
X_RT	3.8180
X_FT	2.0529

**Table 3:** The sensor location DTW distance averages to the rest of the sensors.

Sensor Location Data	Avarage DTW Distance
X_MM	1147.557
X_FM	1228.157
X_RM	1279.843
X_RB	1396.029
X_MT	1606.057
X_RT	1859.757
X_FT	1903.729
X_MB	2084.629

**Table 4:** Sensor locations with the best performance for a given number of sensors.

Number of Sensors	Sensor Location Data	Mean Absolute Errors
1	X_RB	0.9898
1	X_MM	1.0046
2	X_MT X_RB	0.9332
2	X_MT X_RM	0.9390
3	X_FT X_MT X_RM	0.7459
3	X_FT X_MM X_RM	0.7872
4	X_FT X_MT X_MB X_RT	0.6591
4	X_FT X_MT X_MB X_RM	0.6615
5	X_FT X_FM X_MT X_MB X_RM	0.6477
5	X_FT X_FM X_MT X_MM X_RT	0.6599
6	X_FT X_FM X_MT X_MB X_RT X_RB	0.6099
6	X_FT X_FM X_MT X_MB X_RM X_RB	0.6457

## Data Availability

Publicly available datasets were analyzed in this study. This data can be found here: https://data.mendeley.com/v1/datasets/nxttkftnzk/draft?a=7d8b1fed-c1c3-4aa3-8cf3-5b385d221237.

## Abbreviations

The following abbreviations are used in this manuscript:

MDPI	Multidisciplinary Digital Publishing Institute
ML	Machine Learning
WSN	Wireless Sensor Network
ARMA	Auto-Regressive Moving Average
ARIMA	Auto-Regressive Integrated Moving Average
VAR	Vector Autoregression
RNN	Recurrent Neural Network
LSTM	Long Short-Term Memory
GRU	Gated Recurrent Unit
CNN	Convolutional Neural Network
DTW	Dynamic Time Warping
RTL	Real Time Logger
API	Application Programming Interface
MAE	Mean Absolute Error
